# A high-throughput screening strategy for detecting CRISPR-Cas9 induced mutations using next-generation sequencing

**DOI:** 10.1186/1471-2164-15-1002

**Published:** 2014-11-20

**Authors:** Charles C Bell, Graham W Magor, Kevin R Gillinder, Andrew C Perkins

**Affiliations:** Mater Research, Faculty of Medicine and Biomedical Science, The University of Queensland, Woolloongabba, Queensland Australia; Department of Cancer Services, Princess Alexandra Hospital, Brisbane, Queensland Australia

**Keywords:** CRISPR-Cas9, Genome editing, Next-generation sequencing, Screening strategy, CRISPR, Cas9, Indel

## Abstract

**Background:**

CRISPR-Cas9 is a revolutionary genome editing technique that allows for efficient and directed alterations of the eukaryotic genome. This relatively new technology has already been used in a large number of ‘loss of function’ experiments in cultured cells. Despite its simplicity and efficiency, screening for mutated clones remains time-consuming, laborious and/or expensive.

**Results:**

Here we report a high-throughput screening strategy that allows parallel screening of up to 96 clones, using next-generation sequencing. As a proof of principle, we used CRISPR-Cas9 to disrupt the coding sequence of the homeobox gene, *Evx1* in mouse embryonic stem cells. We screened 67 CRISPR-Cas9 transfected clones simultaneously by next-generation sequencing on the Ion Torrent PGM. We were able to identify both homozygous and heterozygous *Evx1* mutants, as well as mixed clones, which must be identified to maintain the integrity of subsequent experiments.

**Conclusions:**

Our CRISPR-Cas9 screening strategy could be widely applied to screen for CRISPR-Cas9 mutants in a variety of contexts including the generation of mutant cell lines for *in vitro* research, the generation of transgenic organisms and for assessing the veracity of CRISPR-Cas9 homology directed repair. This technique is cost and time-effective, provides information on clonal heterogeneity and is adaptable for use on various sequencing platforms.

**Electronic supplementary material:**

The online version of this article (doi:10.1186/1471-2164-15-1002) contains supplementary material, which is available to authorized users.

## Background

The recent development of genome editing techniques, such as Zinc Finger Nucleases, TALENS and CRISPR-Cas9, is revolutionizing the way that molecular biologists interrogate the functionality of the eukaryotic genome. The most exciting and rapidly expanding of these technologies, CRISPR-Cas9 was only developed last year, yet it has already had a huge impact on basic science research, biotechnology and holds huge promise for medicine [1]. CRISPR-Cas9 genome editing is based on the type II CRISPR adaptive immune pathway that is utilized by *Streptococcus pyogenes*, to defend against bacteriophage infection [2, 3]. This simple two-part system consists of a customizable guide RNA that directs the Cas9 endonuclease to a genomic locus, causing double stranded DNA breaks (DSB) [4]. These DSBs are subsequently repaired through either, non-homologous end joining (NHEJ) or homology directed repair (HDR), resulting in the introduction of mutations into the targeted locus [5]. By modifying this endogenous immune pathway, CRISPR-Cas9 genome editing allows the generation of insertion or deletion (indel) mutations, the deletion of large genomic loci, such as entire exons, and the introduction of specific small DNA changes, such as SNPs [4, 6, 7]. Other variant forms of Cas9 protein have also been generated through mutation its catalytic domain. For example, the catalytically inactive version of the Cas9 (dCas9) can be used to interfere with target gene expression, activate or overexpress target genes or visualize genomic loci [8–11]. The relatively high efficiency and broad applicability of this technology has opened the door to experiments that were hitherto not possible. These include genetic engineering in non-model organisms [12], perturbing entire gene families or regulatory networks in a single experiment [7] and correcting disease mutations *in vivo* [13]. These studies have and will continue to provide insights into how the genome is structured, regulated and can be modified to prevent or cure disease.

In particular, CRISPR-Cas9 technology is ideal for genetic engineering of cultured cells, as has been demonstrated by its successful implementation in various studies [5]. The entire experimental process from design to the identification of mutant cell clones can be completed within 4 weeks, at a relatively low cost [6, 14]. The general CRISPR-Cas9 protocol for genetic engineering of cultured cells involves: the design of guide RNAs to target the locus of interest, construction of expression cassettes containing Cas9 and the guide RNA, transfection of the CRISPR-Cas9 construct into cells of interest, selection of transfected-clones (optional), expansion of positive clones in culture and finally screening for the desired CRISPR induced mutations (described in detail in Ran et al. [6]). The most time-consuming, labour intensive and costly stage in the CRISPR-Cas9 methodology involves screening and identification of mutant cell clones.

Currently CRISPR-Cas9 induced mutations are identified through PCR amplification of targeted regions followed by cloning into plasmids and Sanger sequencing, or deep sequencing using amplicon-based kits, such as the Nextera XT DNA sequencing kit [6]. Plasmid cloning and Sanger sequencing is time consuming, laborious, relatively expensive and provides limited information on the heterogeneity of the clone, while the currently available deep sequencing kits are relatively expensive and are specific to particular sequencing platforms.

In this paper we propose a cheap, high-throughput strategy for screening individual clones using next-generation sequencing. We also demonstrate the utility of this approach by identifying mutants for the homeobox gene, *Evx1* in mouse embryonic stem (mES) cells. This method allows the screening of up to 96 clones in a single sequencing run. In our methodology we use the IonTorrent PGM, however this strategy is generalizable and could also be adapted for use on other next-generation sequencing platforms.

## Results and discussion

### Developing a cheap, high-throughput screening strategy for CRISPR-Cas9

**Figure 1:**
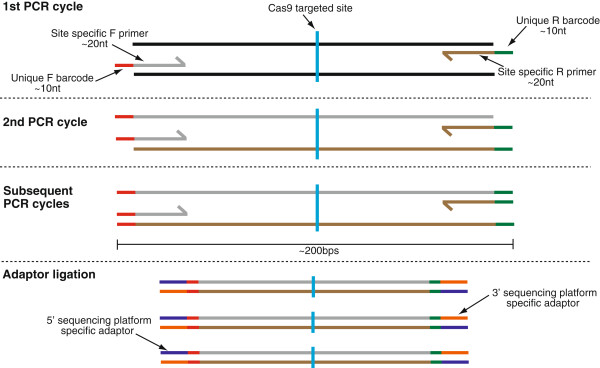
**Barcoded library preparation strategy.** Forward and reverse PCR primers were designed with a unique ~10 nt barcode along with a ~20 nt site specific sequence, which will amplify around the CRISPR-Cas9 targeted site. In the first PCR cycle, either the forward or reverse barcode is added to end of the PCR product. In the second PCR cycle, the opposite barcode is added to each PCR product. In each subsequent cycle, both the forward and reverse barcodes are amplified along with the targeted region. After the PCR, sequencing platform specific adaptors are ligated to the pool of barcoded amplicons in a single reaction.

The preparation of barcoded DNA libraries is generally achieved either through the ligation of unique barcode and adaptor sequences to fragmented DNA [15], or by incorporating barcode and adaptor sequences into PCR primers, so that the barcodes and the appropriate adaptors are added to the PCR product during the amplification process [16]. The ligation strategy is generally utilized when the sample contains a complex pool of DNA fragments, or when the identity of the DNA fragments is unknown, for example in a ChIPseq experiment. Whereas, when the region to be sequenced is small and the number of samples is large, it is practical to incorporate barcodes and adaptors into “fusion” PCR primers. However, “fusion primers” can become prohibitively expensive as the number of samples to be sequenced in parallel increases. Often over ~50-60 nucleotides in length, they require more extensive purification steps to ensure that the majority of the primers are full-length and contain the complete sequencing adaptor sequences. In order to create a more cost-effective amplicon library for multiplexing a large number of CRISPR-Cas9 clones, we used a hybrid approach, in which the DNA barcode is included in the primer, along with a target specific sequence, while the sequencing platform specific adaptors are ligated in a subsequent reaction (Figure 1).

Since the NHEJ repair pathway results in indels of various sizes at the CRISPR-Cas9 targeted site, we reasoned that screening primers should be designed to create an amplicon over the targeted region and cover as much of the surrounding DNA as possible. For the IonTorrent PGM, we decided on an amplicon length of 200 bps, maximizing our ability to detect a variety of mutations, while ensuring that the majority of the reads reach the forward and reverse barcode.

**Figure 2:**
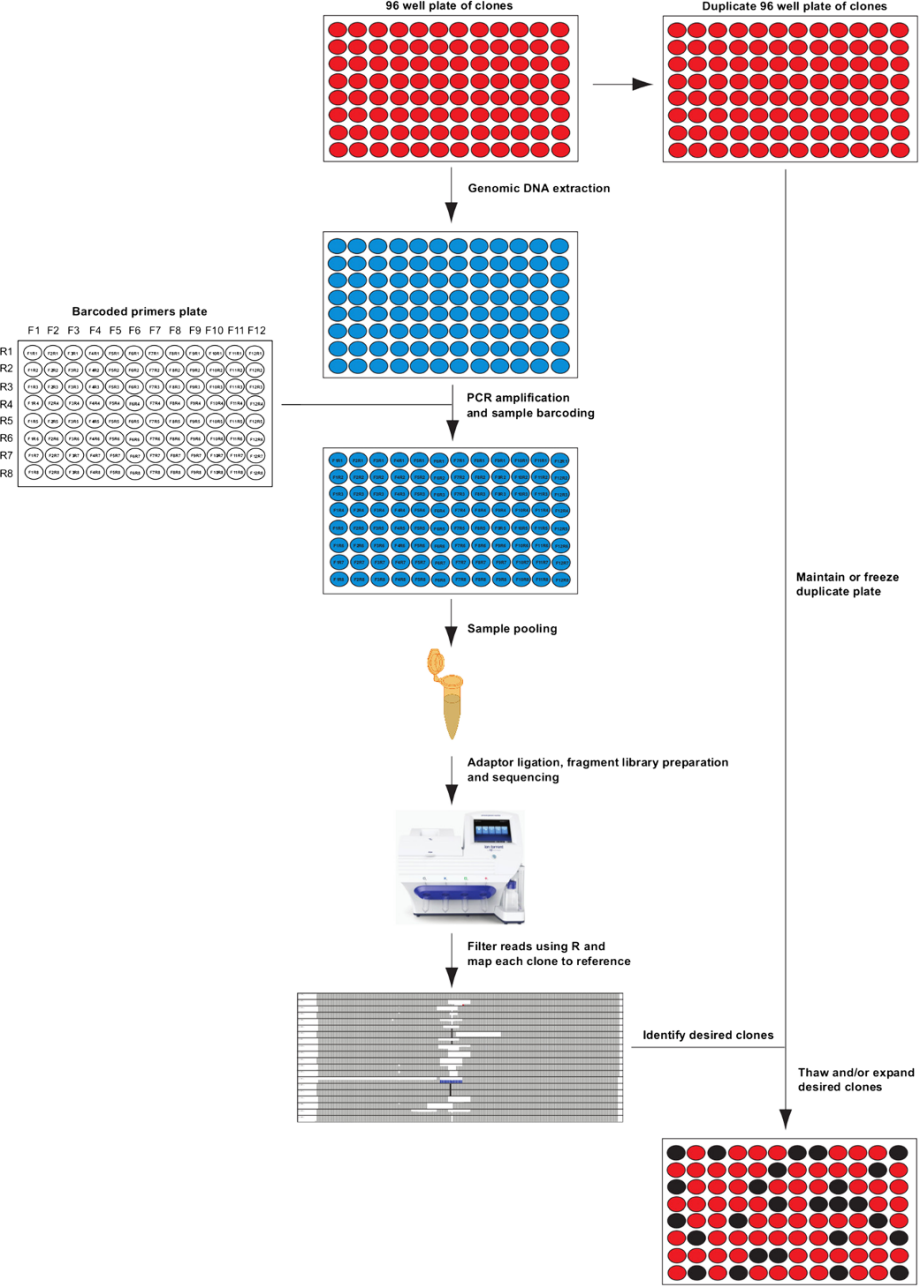
**CRISPR-Cas9 screening workflow.** Duplicate 96 well plates of cell clones are generated. One plate is frozen down or maintained for later use. From the other plate, genomic DNA is extracted, and the targeted region is amplified and uniquely barcoded in each well. The DNA is then pooled, adaptors ligated, and a fragment library is prepared. The sequencing library is then sequenced on the IonTorrent PGM. Reads are filtered and visualized on IGV, to identify the desired clones. The desired clones are then thawed and/or expanded for subsequent experiments.

We used a row and column based barcoding system, to reduce the number of primers required for screening. By using 12 barcoded forward primers (columns) and 8 barcoded reverse primers (rows), it is possible to create a uniquely barcoded amplicon for up to 96 clones (Figure 2). We chose to use the barcode sequences from the published IonXpress barcode set, as they have been optimized to work with the flow set of bases used by the Ion Torrent PGM. Using this barcoding system requires a total of only 20 primers, each approximately 30 nucleotides in length.

### Screening for CRISPR-Cas9 induced mutations in *Evx1*

In order to demonstrate the applicability of our screening strategy, we generated mutant mES cells for the homeobox gene, *Evx1*, which has previously been shown to be dispensable for mouse embryonic development [17]. An overview of our general screening methodology is shown in Figure 2.

After transfection, sorting and expansion of CRISPR-Cas9 targeted clones, we amplified the targeted region of *Evx1* using barcoded forward and reverse primers. We validated the amplification of *Evx1* in a number of clones using one set of barcoded primers (Additional file 1: Figure S1). Using the aforementioned strategy, we then amplified 67 clones, each with a unique barcoded identity, in a 96 well plate format. DNA was pooled from each of the wells in equal proportion, and quantified prior to template library preparation. We neglected to normalise the quantity of DNA from each PCR product, as we reasoned the efficiency of PCR from each clone would likely be similar and that even with discrepancies in the concentration of DNA, we would achieve sufficient sequencing coverage of the least abundant clone to identify the CRISPR induced mutations. Sequencing adaptors (Ion Torrent A and P1) were ligated to the pooled DNA, and the library was then sequenced on the IonTorrent PGM. To determine the mutations present in each clone, it was necessary to de-multiplex the samples. We achieved this by writing a custom script in the R programming language that makes use of the open source ‘ShortRead’ package available from the Bioconductor website (http://www.bioconductor.org/). We applied this script to our data, which produces a separate FastQ file for each individual clone and then mapped all of the FastQ files to mm9 using Bowtie2.

Sufficient coverage was achieved across all 67 clones, enabling the identification of mutations in each clone. The amplicon coverage varied from 313 fold to 6591 fold, with a mean coverage of 2455 fold (Additional file 1: Figure S2). Over 95% of clones were covered between 1/5^th^ and 5 times the mean coverage indicating that our sequencing coverage was fairly uniform and that equalization of the individual PCR products was not required.

**Figure 3:**
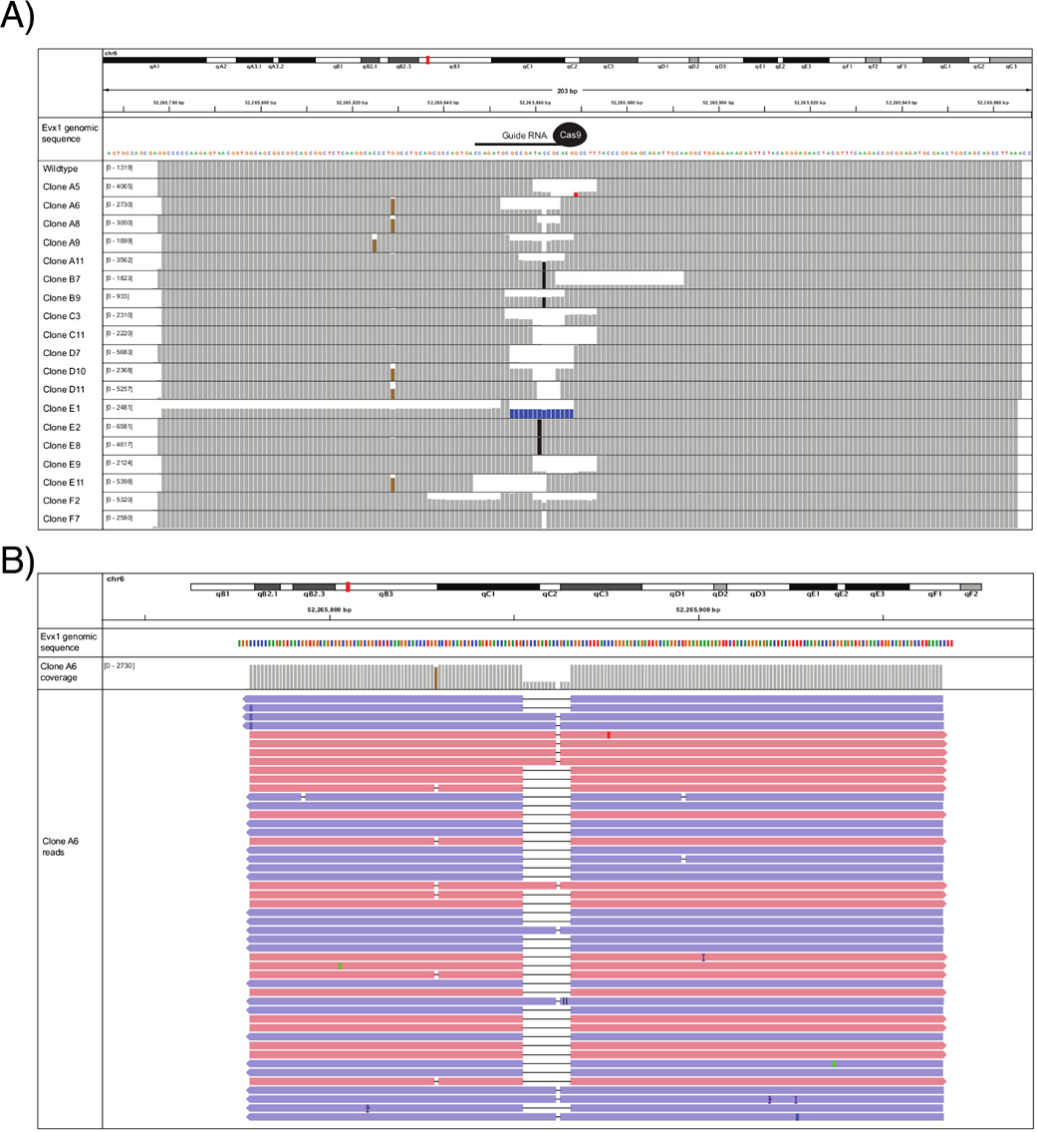
**Visualization of CRISPR-Cas9 mutant clones. A)** Coverage across the barcoded amplicon in a variety of different ‘knockout’ CRISPR-Cas9 clones. The wildtype clone has full coverage over the whole amplicon, while the mutant clones have variable indels around the targeted site. Deletions are represented by a gap in the coverage, insertions are shown as black bars, while substitutions are shown in red. Blue bars represent mismatched mapped reads, due to a large deletion. Brown bars show presumed sequencing errors, which each have significant strand bias and commonly occur at the same position in multiple samples. **B)** The sequencing reads and coverage for clone A6, which is a compound heterozygote with a 1 bp deletion on one allele and a 13 bp deletion on the other allele. The deletions at the targeted site are present at equal proportion on both strands (blue and red), whereas the sequencing error upstream is present only on the red strand.

We visualized the data using Integrated Genomics Viewer (IGV) version 2.3.34 and annotated the mutations by visual inspection. Sequencing errors could easily be distinguished from real indels by the fact that they show an extreme strand bias and typically occur in the same position in multiple samples [18, 19] (Figure 3). *Bona fide* mutations generally map in roughly equal proportions to both strands (Figure 3B).

As has been reported previously, the incidence of CRISPR-Cas9 induced mutations was very high (65 out of 67 clones showing some form of genetic insult) and all mutations were located close to the targeted sequence [7] (Figure 3A). Our technique allowed us to distinguish heterozygous mutants from homozygous mutants, as well as identify samples that contained more than two different types of mutations indicating a mixed clone. Overall we identified 10 homozygous mutants, 27 compound heterozygotes, 4 heterozygotes, 2 wildtypes, and 21 clones that showed more than two different alleles (3 clones could not be mapped).

We suspect that the mixed clones are likely the result of sorting multiple cells into a single well during the FACS process or ongoing pCas9 activity after cell division, resulting in different mutations in each daughter cell. Importantly, using our screening method, we were able to identify the presence of mixed clones, which could be missed when screening using plasmid-cloning and Sanger sequencing. Establishing the integrity of the derived clones is essential for downstream analysis, especially when the desired result is the complete disruption of the targeted gene. Once identified, mixed clones can either be avoided in subsequent functional assays, or clonally isolated by serial dilution.

## Conclusions

We developed a cheap, high-throughput screening strategy for identifying CRISPR-Cas9 induced mutations using next-generation sequencing. We successfully applied our strategy to identify mES cell clones with a variety of different mutations in the homeobox gene, *Evx1*, using the IonTorrent PGM. We found the frequency of CRISPR-Cas9 induced mutations to be very high and were able to identify 37 clones in which both alleles of the *Evx1* were disrupted. Our methodology has numerous advantages over the current screening strategies. Firstly, it is cost effective, as it requires only 20, 30 nt primers to uniquely barcode up to 96 clones, additionally, as the coverage requirements to identify CRISPR-Cas9 induced mutations are quite low, it is possible to multiplex with additional samples. Secondly, this method is generalizable, opening the door for deep sequencing-based mutational screening on a variety of sequencing platforms. Finally, our strategy provides information on the heterogeneity of each clone (mixed clones), which could be missed when screening with plasmid cloning and Sanger sequencing.

Although in this study we have focused on screening murine cells in culture, we envision that our strategy could be widely applied to screen for CRISPR-Cas9 mutants, in a variety of contexts. Our strategy could be adapted to screen for mutants in CRISPR-Cas9 transgenic organisms. It is also particularly well-suited for screening mutations in polyploid organisms or cell lines, as next-generation sequencing provides a global picture of all alleles. This methodology provides a further step forward in the rapidly expanding field of CRISPR-Cas9 genome editing.

## Methods

### Cell culture

W9.5 mES cells were routinely maintained in ES cell media with LIF (1000 U/ml) on gelatinized tissue culture plates at 37°C and 5% CO_2_.

### CRISPR-Cas9 design and cloning

A guide RNA was designed to target the second exon of *Evx1*, using the CRISPR-Cas9 design tool (crispr.mit.edu.au). This guide was cloned into pCas9 (BB)2A-GFP (Addgene PX458), as described previously [6].

### Transfection, sorting and expansion of clones

W9.5 mES cells were electroporated using the BioRad GenePulser II with pCas9-*Evx1*-2A-GFP plasmid according to the manufacturer’s instructions. After 24 hours, mES cells were dissociated with trypsin and FACS sorted on the MoFlow Astrois Cell Sorter to enrich for GFP + cells. We gated for cells that had high GFP expression (5% of the original cell population) and sorted individual cells into 3 gelatinized 96 well plates, to allow for cell death in the sorting and expansion process. Sorted cells were expanded for a week and inspected to ensure that cells were present. Two duplicate 96 well plates were generated from the surviving clones, by transferring half of the clone to one 96 well plate and the remaining half to another 96 well plate in the corresponding well. Once the clonal cells were ~80% confluent in each 96 well plate, genomic DNA was extracted from one of the plates using a simple plate DNA extraction (outlined below). Cells in the duplicate 96 well plate were frozen down in 10% DMSO and stored at −80°C.

### Plate genomic DNA extraction

Adherent cells in a 96 well plate were washed with PBS. PBS was discarded and 50ul of lysis buffer (25 mM NaOH, 0.2 mM EDTA) was added to the cells. After 5 minutes of rotating on an orbital shaker, cells were transferred to a 96 well PCR plate and heated at 95°C for 30 minutes. After heating, 50 ul of Tris buffer (Tris–HCl 40 mM) was added to make a final volume of 100 uL of genomic DNA.

### Barcoded amplicon primer design and validation

Forward and reverse primers were designed to *Evx1*, 100 base pairs either side where the guide RNA was designed. Unique barcodes were added to 12 forward and 8 reverse primers to allow the unique barcoding of an entire 96 well plate (Figure 1). Barcode sequences were taken from the published IonXpress barcode set.

### Sample barcoding and PCR amplification

5 uL was transferred from each well of the genomic DNA plate to another 96 well PCR plate. 0.2 μM of the barcoded forward and reverse primers to amplify the targeted *Evx1* locus were added to each well, along with 1.5 mM MgCl_2_, 0.2 mM dNTPs, 10X PCR buffer (−MgCl_2_) and platinum Taq DNA polymerase (Invitrogen). PCR was then conducted in the plate for 40 cycles (95°C-2 min, 40 cycles of (95°C-30 sec, 55°C-30 sec, 72°C-30 sec), 72°C-30 sec).

### Library preparation

PCR products were combined in equal proportion and the combined pool of PCR products was purified in a single tube using Ampure XP reagent (#A63881, Beckmann-Coulter) at 1.8X sample concentration, as per the manufacturer’s instructions. Sequencing libraries were created by ligating adaptor sequences to the pooled DNA using the Ion Plus Fragment Library Kit (#4471252, Life Technologies). Briefly, 50 ng of purified PCR product was end-repaired and then A and P1 adaptors were ligated. Amplicons with adaptors were purified with Ampure XP reagent at 1.2X sample concentration. Final library concentration was measured using the Qubit fluorometer (#Q32866, Invitrogen) and the double-stranded DNA high-sensitivity assay kit (#Q32854, Invitrogen).

### DNA sequencing

Libraries were diluted to 22 ng/mL (~100 pM) and sequencing template was prepared using the Ion PGM Template OT2 200 kit (#4480974, Life Technologies) on the Ion One Touch 2 instrument according to manufacturer’s instructions. Templated Ion Sphere Particles (ISPs) were enriched on an Ion One Touch ES and sequencing was performed on an Ion Torrent PGM along with the Ion PGM Sequencing 200 Kit v2 (#4482006, Life Technologies).

### Sequence mapping

FastQ reads were mapped to the *Mus musculus* genome version 9 (mm9) using Bowtie2 with default parameters, apart from an adjustment to relax the gap extension penalty (option: −-rdg 5,1). De-multiplexing of barcoded samples was performed in R using a custom script (Additional file 2).

### Ethics statement

No human tissue or patient information was obtained for use in this study. All work was conducted using established and commercially available mouse embryonic stem cells (w9.5). This study was conducted according to the Australian Code of Responsible Conduct of Research.

### Availability of supporting data

The data sets supporting the results of this article are available in the LabArchives, LLC, at http://dx.doi.org/10.6070/H4RB72KT.

## Electronic supplementary material

Additional file 1: **Contains Supplementary Figures S1-2.** (PDF 366 KB)

Additional file 2: **Contains R-script for de-multiplexing sequencing data.** (TXT 2 KB)
