# Supplementary material for: A high-throughput screening strategy for detecting CRISPR-Cas9 induced mutations using next-generation sequencing
Source: BMC Genomics. 2014 Nov 20;15(1):1002. doi: 10.1186/1471-2164-15-1002 (PMC4246457; doi:10.1186/1471-2164-15-1002)
Supplement: Supplementary file 1 — Additional file 1: Contains Supplementary Figures S1-2. (PDF 366 KB) [file 12864_2014_6673_MOESM1_ESM.pdf]

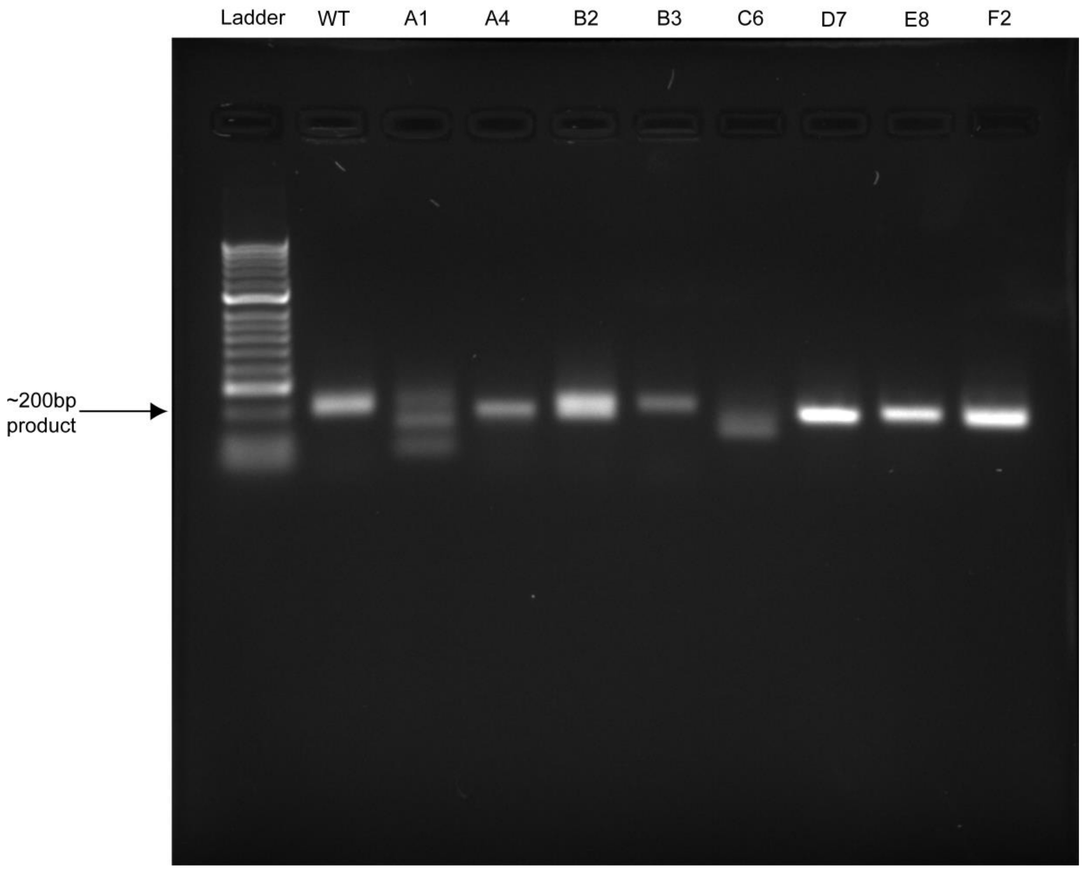

Figure S1: Validation of Evx1 screening primers. The desired ~200bp *Evx1* amplicon can be observed in the wildtype clone (lane 2). CRISPR-Cas9 induced deletions can be observed in other clones by a reduction in PCR product size.

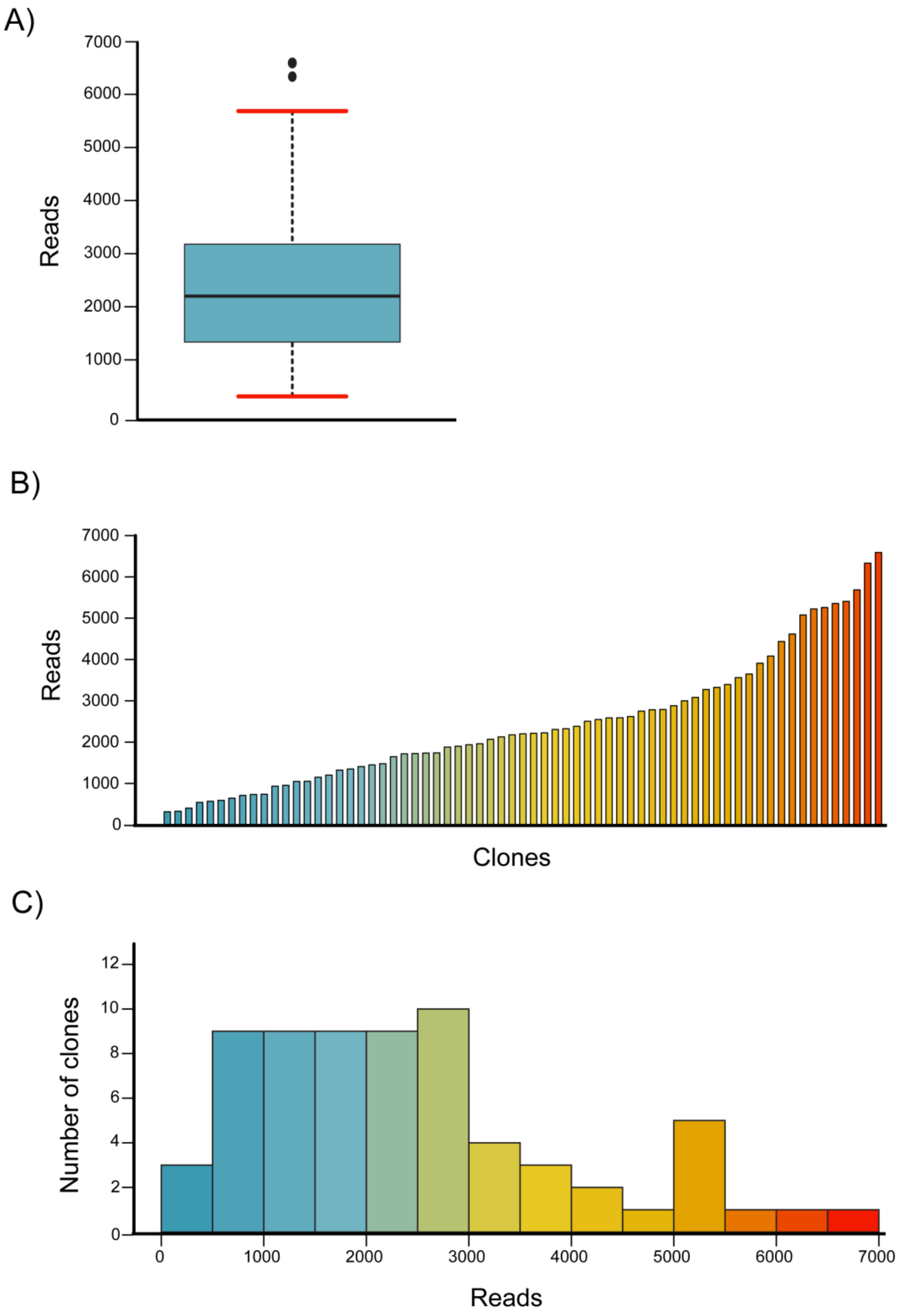

Figure S2: Coverage statistics for CRISPR-Cas9 screening sequencing. **A)** Box and whisker plot demonstrating the relatively even sequencing coverage of each clone. The majority of clones have coverage between ~1200 and ~3100 fold. **B)** Sequencing coverage for each individual clone. **C)** Histogram of sequencing coverage for each clone.
